# Supplementary material for: Oceanographical Context of the First Bloom of the Silicoflagellate Octactis speculum (Ehrenberg) Recorded to Cause Salmon Mortality in a Galician Ria: Was This Bloom a Rare Event in the Iberian Coast?
Source: Toxins (Basel). 2023 Jul 2;15(7):435. doi: 10.3390/toxins15070435 (PMC10467100; doi:10.3390/toxins15070435)
Supplement: Supplementary file 1 [file toxins-15-00435-s001.zip › toxins-2441337-supplementary.pdf]

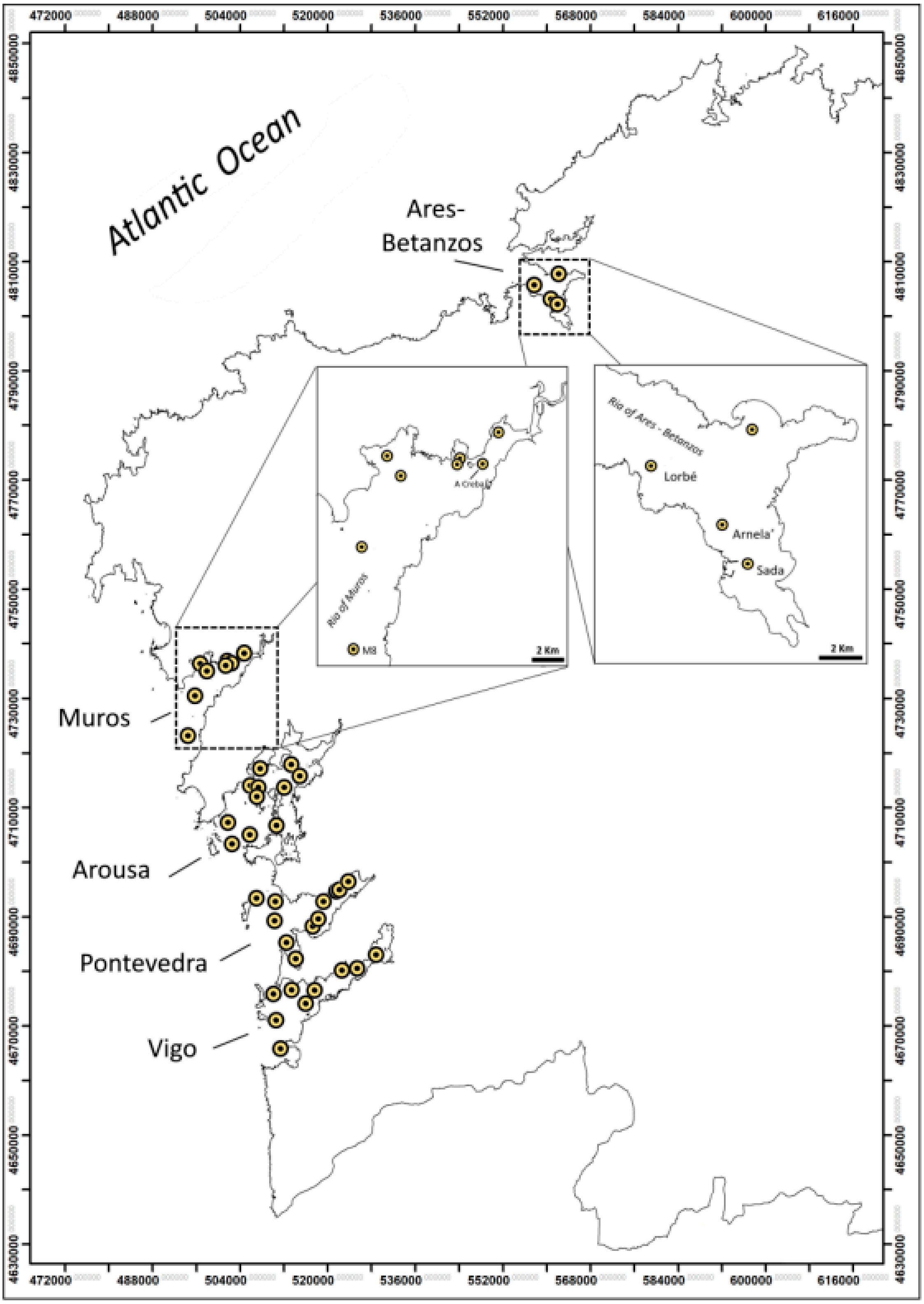

Figure S1. Distribution of the network of oceanographic stations of the INTECMAR (http://www.intecmar.gal/Ctd/Default.aspx) in the Galician coast (NW Iberian Peninsula), with detail of the rias of Ares-Betanzos and Muros.
